# Deficiency of NLRP3 protects cerebral pericytes and attenuates Alzheimer’s pathology in tau-transgenic mice

**DOI:** 10.3389/fncel.2024.1471005

**Published:** 2024-10-30

**Authors:** Wenqiang Quan, Yann Decker, Qinghua Luo, Axel Chemla, Hsin-Fang Chang, Dong Li, Klaus Fassbender, Yang Liu

**Affiliations:** ^1^Department of Clinical Laboratory, Tongji Hospital, Tongji University Medical School, Shanghai, China; ^2^Department of Neurology, Saarland University, Homburg, Germany; ^3^Department of Neurology, The Second Affiliated Hospital of Nanchang University, Nanchang, China; ^4^Cellular Neurophysiology, Center for Integrative Physiology and Molecular Medicine (CIPMM), Saarland University, Homburg, Germany; ^5^Center for Gender-Specific Biology and Medicine (CGBM), Saarland University, Homburg, Germany

**Keywords:** Alzheimer’s disease, cerebrovascular disorders, NLRP3, pericyte, tau

## Abstract

**Introduction:**

Activation of NLRP3-containing inflammasome, which is responsible for IL-1β maturation, has been shown to contribute to Alzheimer’s disease (AD)-associated pathogenesis in both APP- and tau-transgenic mice. However, effects of NLRP3 on pericytes and subsequent cerebrovascular pathology in AD remain unknown.

**Methods:**

NLRP3-deficient and wild-type AD animal models were generated by crossing human P301S tau-transgenic mice and *Nlrp3* knockout mice. AD-associated neuroinflammation, tauopathy, vasculature and pericyte coverage in the brain were investigated using immunohistological and molecular biological methods. To investigate how NLRP3 regulates pericyte activation and survival, pericytes from the brains of *Nlrp3* knockout and wild-type mice were cultured, treated with IL-1β and H_2_O_2_ at different concentrations and analyzed by confocal microscopy and flow cytometry after staining with fluorescently labelled phalloidin, annexin-V and PDGFRβ antibody.

**Results:**

Deficiency of NLRP3 (1) reduced Iba-1, GFAP and AT8 antibody-immunoreactive phosphorylated tau-positive cells, without significantly altering transcription of inflammatory genes, (2) preserved cerebral vasculature and pericyte coverage and up-regulated *Osteopontin* gene transcription, and (3) improved cognitive function in tau-transgenic mice. In cell culture, NLRP3 deficiency prevented pericyte apoptosis. Treatment with IL-1β or H_2_O_2_ increased the expression of PDGFRβ in NLRP3-deficient pericytes, but decreased it in NLRP3 wild-type pericytes in a dose-dependent manner.

**Discussion:**

Inhibition of NLRP3 can promote pericyte survival, improve cerebrovascular function, and attenuate AD pathology in the brain of tau-transgenic mice. Our study supports NLRP3 as a novel therapeutic target for Alzheimer’s patients.

## Introduction

In addition to extracellular amyloid-*β* (Aβ) deposits, intracellular neurofibrillary tangles and neuroinflammation, vascular dysfunction has been recognized as a new pathogenic factor for Alzheimer’s disease (AD; Scheltens et al., 2016; Strickland, 2018). For example, postmortem brain tissue from up to 80% of diagnosed AD patients exhibits vascular pathologies ranging from large artery atherosclerosis and cerebral amyloid angiopathy to microvascular disorders and impairment of blood–brain barrier (BBB; Cortes-Canteli and Iadecola, 2020). However, how brain vessels are damaged remains unclear.

Pericytes, enwrapping the endothelial cells of brain capillaries, regulate BBB permeability, angiogenesis, hemodynamic responses and neuroinflammation (Sweeney et al., 2016). Pericyte dysfunction may contribute to cerebral vascular damage. In fact, the number of pericytes decreases in AD patients in correlation with increased BBB permeability (Ding et al., 2020; Sengillo et al., 2013). Pericytes express platelet-derived growth factor receptor *β* (PDGFRβ), which is shed by the pericytes once the cells are injured. Soluble PDGFRβ has been reported to increase in cerebrospinal fluid at a very early stage in old adults with cognitive deficits (Nation et al., 2019). In addition, oligomeric Aβ induces pericyte contraction and blocks microcirculation by triggering the release of reactive oxygen species and endothelin-1 in the brain (Nortley et al., 2019). APOE4 expression promotes pericyte injury and BBB impairment in the hippocampus and medial temporal lobe of AD patients (Montagne et al., 2020). In mouse models with a loss-of-function mutation in *Pdfgrβ* gene, pericytes are lost during aging, which reduces microvessel length and blood flow, increases BBB permeability and eventually leads to neurodegeneration (Bell et al., 2010). Thus, dysfunctional pericytes impact cerebral vascular pathology. However, the mechanisms mediating pericyte responses to extracellular stimuli and subsequent cellular impairment in the AD brain are still largely unknown.

Pericytes express pattern recognition receptors, such as Toll-like receptor (TLR)-2 and -4, and NLR family pyrin domain containing (NLRP)-1 and -3 (Guijarro-Munoz et al., 2014; Leaf et al., 2017; Nyul-Toth et al., 2017; Quan et al., 2020), and respond to inflammatory stimuli, e.g., interleukin (IL)-1β and tumor necrosis factor (TNF)-*α* (Quan et al., 2020; Smyth et al., 2018). We hypothesized that innate immune receptors regulate the activation and damage of pericytes in AD. Deficiency of NLRP3 has been shown to prevent disease progression in both APP- and tau-transgenic mice (Heneka et al., 2013; Ising et al., 2019; Jiang et al., 2021); however, the effects of NLRP3 on microvascular circulation in AD brains were not investigated. We recently observed that NLRP3 deficiency decreases the vasculature and pericyte coverage on vessels in 9-month-old mouse brain under a physiological condition (Quan et al., 2020). We wondered whether NLRP3 regulated pericyte pathology in AD mice. We cross-bred tau-transgenic mice and *Nlrp3*-knockout mice and investigate the effects of NLRP3 on pericytes and vasculature in AD mice. To investigate the effect of NLRP3 specifically on pericytes, we cultured NLRP3-deficient and wild-type pericytes and analyzed their responses to the inflammatory mediators IL-1β and H_2_O_2_.

## Materials and methods

### Animal models and cross-breeding

*Nlrp3* gene knockout (NLRP3^−/−^) mice on C57BL/6 genetic background were kindly provided by N. Fasel (University of Lausanne, Lausanne, Switzerland; Martinon et al., 2006). P301S tau-transgenic (tau^tg^) mice were bought from the Jackson Laboratory, Bar Harbor, MA, United States [B6;C3-Tg(Prnp-MAPT*P301S)PS19Vle/J; Stock number: 008169], which over-express human tau mutant (P301S) under the direction of mouse prion protein promoter (Yoshiyama et al., 2007). Tau^tg^ mice had been back-crossed with C57Bl/6 J mice for ≥6 generations before starting the current project. NLRP3^−/−^ mice were cross-bred with tau^tg^ mice to obtain tau^tg^Nlrp3^−/−^ and tau^tg^Nlrp3^+/+^ genotypes. Animal experiments were conducted in accordance with national rules and ARRIVE guidelines, and authorized by Landesamt für Verbraucherschutz, Saarland, Germany (registration numbers: 16/2018) and Tongji University, China.

### Morris water maze

The Morris water maze test was used to assess the cognitive function of tau^tg^ mice and their wild-type littermates, as previously described (Schnoder et al., 2023). Mice were trained to find the hidden escape platform. There were four trials per training day; with a trial interval of 15 min. Latency time, path length, and velocity were recorded with Ethovision video tracking equipment and software (Noldus Ethovision, Wageningen, The Netherlands). After 6 training days, there were 1 day of rest, and a probe trial on the 8th day. During the probe trial, the platform was removed, and the swimming path was recorded for 5 min. The frequency of entries in the location of original platform were measured.

### Tissue collection

Animals were euthanized by inhalation of overdose isofluorane and perfused with ice-cold phosphate-buffered saline. The left hemisphere was fixed in 4% paraformaldehyde (PFA) (Sigma-Aldrich Chemie GmbH, Taufkirchen, Germany) for histological analysis and the right hemisphere was homogenized in Trizol for isolation of RNA using our routine protocol (Quan et al., 2021).

### Histological analysis

Brain tissues were embedded in paraffin after dehydration. To quantify vasculature in the brain, our established protocol was used (Quan et al., 2021; Quan et al., 2020). Thirty-μm-thick sagittal sections were serially cut from the left hemisphere. Four serial sections per mouse with 300 μm of distance in between were deparaffinized, heated at 80°C in citrate buffer (10 mM, pH = 6) for 1 h and digested with Digest-All 3 (Pepsin; Thermo Fisher Scientific, Darmstadt, Germany) for 20 min. After blocking with 0.2% casein in PBS/0.3% Triton X-100, brain sections were stained with rabbit anti-collagen IV polyclonal antibody (Catalog-No: # ab6586; Abcam, Cambridge, United Kingdom) and Alexa488-conjugated goat anti-rabbit IgG (Thermo Fisher Scientific). After being mounted, the whole hippocampus was imaged with Microlucida (MBF Bioscience). The length and branching points of collagen type IV staining-positive blood vessels were analyzed with a free software, AngioTool^1^ (Zudaire et al., 2011). The parameters of analysis for all compared samples were kept constant. The length and branching points were adjusted with area of interest.

To analyze pericyte coverage in capillaries, four serial sections were deparaffinized, digested and blocked as described above. Brain sections were incubated with rabbit anti-PDGFRβ monoclonal antibody (clone: 28E1; Cell Signaling Technology Europe, Frankfurt am Main, Germany) at 4°C overnight and then Alexa488-conjugated goat anti-rabbit IgG (Thermo Fisher Scientific) at room temperature for 1 h. Thereafter, brain sections were further stained with biotin-labeled *Griffonia Simplicifolia* Lectin I isolectin B4 (Catalog-No: B-1205; Vector Laboratories, Burlingame, CA, United States) and Cy3-conjugated streptavidin (Roche Applied Science, Mannheim, Germany). Fluorescence-labeled areas in the hippocampus were measured with Image J software.^2^ The coverage of pericytes was calculated as a ratio of PDGFRβ/isolectin B4-positive staining area.

To count microglia and astrocytes, serial brain sections were prepared as vessel staining but without pepsin digestion, and stained with rabbit anti-ionized calcium-binding adapter molecule (Iba)-1 antibody (1:500, Cat.-No, 019–19741; Wako Chemicals, Neuss, Germany), or rabbit anti-glial fibrillary acidic protein (GFAP) antibody (1:500, Code-No, Z0334, Agilent Technologies Deutschland GmbH, Waldbronn, Germany). Thereafter, tissues were incubated with biotin-conjugated goat anti-rabbit IgG and Cy3-conjugated streptavidin (Thermo Fisher Scientific). Iba-1 or GFAP-positive cells were counted with Optical Fractionator as we did previously (Liu et al., 2014) on a Zeiss AxioImager.Z2 microscope equipped with a Stereo Investigator system (MBF Bioscience, Williston, VT, United States).

To evaluate tau pathology in tau^tg^ mice, four serial sections were chosen. Brain tissues were stained according to our established protocols (Schnoder et al., 2023) with a mouse monoclonal antibody against human phospho-tau (Ser202, Thr205) (5 μg/mL, clone, AT8, Thermo Fisher Scientific). To stained apoptotic cells, rabbit monoclonal antibody against cleaved caspase-3 (Asp175) (clone: 5A1E; Cell Signaling Technology Europe) was used. Because of low numbers of immunoreactive cells, we did not use stereological analysis, but counted labeled cells in the whole brain region. Data were recorded as the number of labeled cells divided by the full area (in square millimeters) of interest.

In additional experiments, we performed immunofluorescent staining of brain tissues for lipid peroxidation with rabbit antibody against 4-hydroxy-2-nonenal (4HNE; Cat.-No: HNE11-S; Alpha Diagnostic Intl. Inc., San Antonio, Texas, United States). To better present the images on the fluorescent staining, stack images were acquired using a 40× objective with an interval of 2 μm for 5 layers, and Z-projected with maximum intensity.

### Quantitative PCR for measurement of gene transcripts

Total RNA was isolated from mouse brains with TRIzol and reverse-transcribed. Gene transcripts were quantified with established protocols (Hao et al., 2016; Quan et al., 2021) and Taqman gene expression assays of mouse *Tnf-α*, *Il-1β*, *Chemokine* (*C–C motif*) *ligand 2* (*Ccl-2*), *Il-10*, *Chitinase-like 3 (Chi3l3)*, *Mannose receptor C type 1 (Mrc1)*, *Insulin-like growth factor 1* (*Igf1*) and *Gapdh* (Thermo Fisher Scientific). The transcription of *Osteopontin* (*Opn*), and *Vascular endothelial growth factor* (*Vegf*) genes was evaluated using the SYBR green binding technique with the following pairs of primers: *Opn*, 5´-CAGCCATGAGTCAAGTCAGC-3′ and 5´-TGTGGCTGTGAAACTTGTGG-3′; and *Vegf*, 5´-CCCTTCGTCCTCTCCTTACC-3′ and 5´-AGGAAGGGTAAGCCACTCAC-3′.

### Pericytes culture

Pericytes were prepared from 6-week-old NLRP3^−/−^ and NLRP3^+/+^ mice with published protocols (Mehra et al., 2020). Briefly, the cortex and hippocampus were homogenized in HEPES-contained Hanks’ balanced salt solution (HBSS) and centrifuged at 3,000 g in HEPES-HBSS buffer supplemented with dextran from *Leuconostoc* spp. (molecular weight ~ 70,000; Sigma-Aldrich) to delete myelin. Vessel fragments were re-suspended in HEPES-HBSS buffer supplemented with 0.1% bovine serum albumin (Sigma-Aldrich) and filtered by nylon mesh filter. The filtrates passing through 100 but not 20 μm-meshes were digested by Collagenase B, Dispase II and Dnase I (Sigma-Aldrich). The single cell suspentions were cultured on Matrigel matrix (Catalog-No: 354234; BD Biosciences, Heidelberg, Germany)-coated 6-well plate and in Dulbecco’s Modified Eagle Medium (DMEM) contaning 20% calf serum, 2 mM glutamine, 50 μg/mL gentamycin, 1% vitamins, 2% amino acids Basal Medium Eagle (BME) in Basal DMEM media (all culture medium components were bought from Sigma-Aldrich) and 1 ng/mL recombinant mouse basic fibroblast growth factor (bFGF; Catalog-No: 579606; BioLegend, San Diego, CA, United States). Seven days later, pericytes were cultured in Collegen I (Catalog-No: A1048301; Thermo Fisher Scientific)-coated 6-well plate in pericyte culture medium (Catalog-No: 1231; Sciencell Research Laboratories, Carlsbad, CA, United States). The medium was changed every 3 days until experiments.

### Pericyte treatment and analysis

Pericytes cultured in Collagen I-coated 24-well plate at 1.0 × 10^5^ cells/well were treated with recombinant mouse IL-1β (R&D Systems, Inc. Minneapolis, MN, United States) at 0, 5, 10 and 50 ng/mL, or hydrogen peroxide H_2_O_2_ (Sigma-Aldrich Chemie GmbH) at 0, 100 and 500 μM for 24 h. Pericytes were detached by trypsin–EDTA and stained with APC-conjugated annexin V (Catalog-No: 640920; BioLegend) and propidium iodide (Thermo Fisher Scientific), or APC-conjugated recombinant antibody against mouse PDGFRβ (Clone: REA634; Miltenyi Biotec B.V. & Co. KG, Bergisch Gladbach, Germany). Percentage and mean fluorescence intensity (mFI) of pericytes were detected by BD FACSCanto™ II flow cytometry (BD Biosciences).

For confocal imaging, pericytes were cultured on Collagen I-coated cover slip and treated the same as described above. Cells were fixed in 1% PFA, permeabilized with 0.2% Triton-100 and blocked with 0.2% casein in PBS, and then stained with Alexa488-conjugated phalloidin (Thermo Fisher Scientific) for filamentous actin and 4′,6-diamidino-2-phenylindole (DAPI; Sigma-Aldrich Chemie GmbH) for nucleu. Stained pericytes were imaged under LSM 780, Zeiss confocal microscope (Oberkochen, Germany).

### Statistics

Data was presented as mean ± SEM for mice and mean ± SD for cells. For multiple comparisons, one-way or two-way ANOVA followed by Bonferroni or Tukey *post-hoc* test. All statistical analyses were performed with GraphPad Prism 5 version 5.01 for Windows (GraphPad Software, San Diego, CA, United States). Statistical significance was set at *p* < 0.05.

## Results

### NLRP3 deficiency reduces AD pathology in the brain and improves cognitive function in tau-transgenic mice

**Figure 1:**
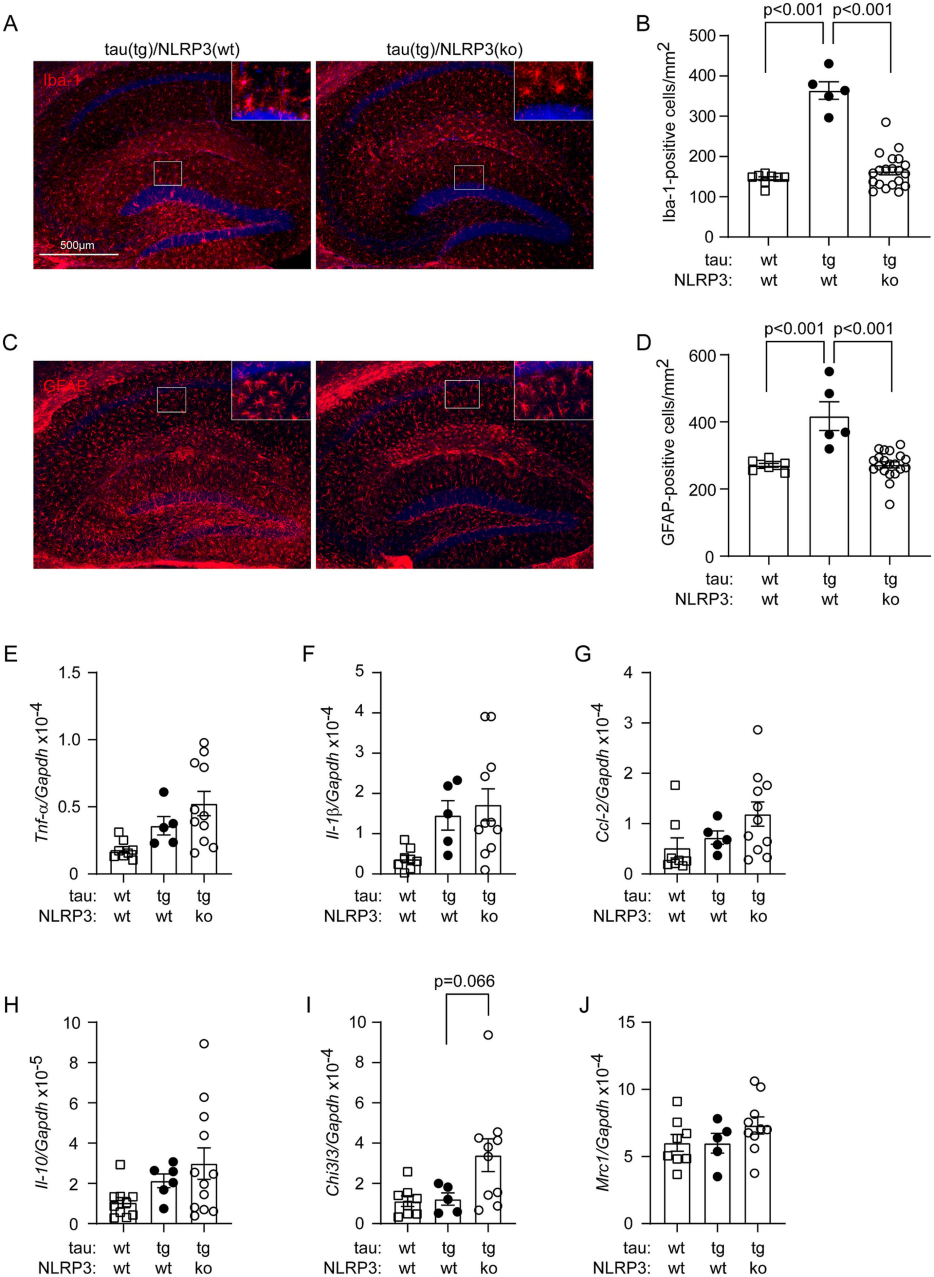
Deficiency of NLRP3 reduces Iba-1- and GFAP-positive cells in the hippocampus of tau-transgenic mice. Nine-month-old tau^tg^Nlrp3^+/+^ (NLRP3 wt) and tau^tg^Nlrp3^−/−^ (NLRP3 ko) mice, and tau wild-type littermates were examined for neuroinflammation. Microglia and astrocytes were counted after immunofluorescent labeling with Iba-1 and GFAP antibodies, respectively (A,C). The brain area-adjusted number of both tested cells in the hippocampus was significantly lower in tau(tg)/Nlrp3(ko) mice than in tau(tg)/Nlrp3(wt) mice (B,D; one-way ANOVA followed by Bonferroni *post-hoc* test, *n* = 5 and 20 for NLRP3 wt and ko tau-transgenic mice, respectively). Compared to tau(tg)/Nlrp3(wt) mice, the numbers of glial cells were also lower in tau(wt)/Nlrp3(wt) mice (B and D; one-way ANOVA followed by Bonferroni *post-hoc* test, *n* = 8 for tau(wt)/Nlrp3(wt) mice). The transcription of inflammatory genes, *Tnf-α*, *Il-1β*, *Ccl-2*, *Il-10*, *Chi3l3* and *Mrc1*, was further measured in the brains of all three mouse groups using real-time PCR (E–J). Knockout of *Nlrp3* gene did not alter the transcription of all tested, with the exception that the transcription level of *Chi3l3* gene tended to be higher in NLRP3-ko tau mice than in NLRP3 wt controls (I; one-way ANOVA followed by Bonferroni *post-hoc* test, n = 5–11 per group).

Deficiency of NLRP3 has been shown to reduce caspase-1 activation and tau pathology, and improve cognitive function in tau-transgenic mice (Ising et al., 2019); however, without addressing its effects on vasculature. We have recently observed that the vasculature decreases in tau-transgenic mice (Decker et al., 2018) and NLRP3 is involved in the homeostasis of vasculature under a physiological condition (Quan et al., 2020). We asked whether NLRP3 regulates vasculature under tauopathy. We cross-bred tau-transgenic mice with *Nlrp3* ko mice. It was not surprising that tau-transgenic expression increased the numbers of Iba-1-positive microglia/brain macrophages (Figures 1A,B; one-way ANOVA, *p* < 0.001) and GFAP-positive astrocytes (Figures 1C,D; one-way ANOVA, *p* < 0.001) in the hippocampus, compared with tau wild-type mice. Knockout of *Nlrp3* gene decreased the numbers of these two cell types in tau-transgenic mice, particularly, in the dentate gyrus (Figures 1A–D; one-way ANOVA, *p* < 0.001). It was surprising that knockout of *Nlrp3* gene did not alter the transcription of the tested inflammatory genes, *Tnf-α*, *Il-1β*, *Ccl-2*, *Il-10* and *Mrc1* (Figures 1E–H,J; one-way ANOVA, *p* > 0.05), with the exception that the transcription level of *Chi3l3* gene tended to be higher in NLRP3-deficient tau mice than in NLRP3 wild-type controls (Figure 1I; one-way ANOVA followed by Bonferroni *post-hoc* test, *p* = 0.066). This result suggested the complexity of inflammatory regulation mediated by NLRP3 deficiency, which is consistent with recent studies showing that: (1) knockout of *Nlrp3* gene does not change the inflammatory signature of microglia in APP/PS1-transgenic mice (Srinivasan et al., 2024); and (2) knockout of *Nlrp3* gene does not reduce plasma IL-18 cytokine in P301S tau-transgenic mice (Paesmans et al., 2024).

**Figure 2:**
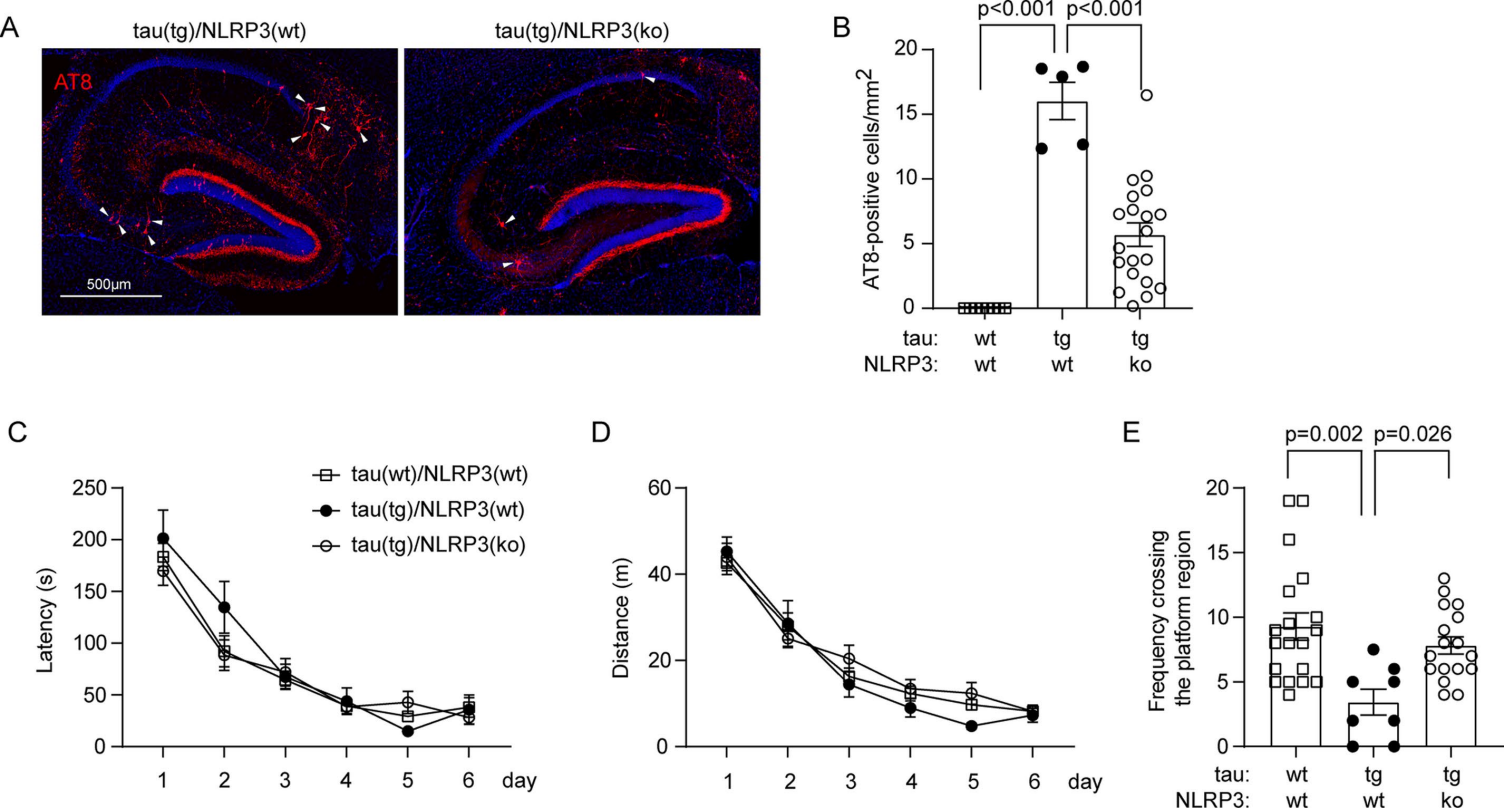
Deficiency of NLRP3 reduces p-tau-positive brain cells and improves cognitive function in tau-transgenic mice. Nine-month-old tau^tg^Nlrp3^+/+^ (NLRP3 wt) and tau^tg^Nlrp3^−/−^ (NLRP3 ko) mice, and tau wild-type littermates were examined for tauopathy. P-tau-positive cells were counted after immunofluorescent labeling with AT8 antibody (A). The brain area-adjusted number of AT8-positive cells in the hippocampus was significantly lower in tau(tg)/Nlrp3(ko) mice than in tau(tg)/Nlrp3(wt) mice (B; one-way ANOVA followed by Bonferroni *post-hoc* test, *n* = 5 and 20 for NLRP3 wt and ko tau-transgenic mice, respectively). The water maze test was used to assess the cognitive function of the mice. The three groups of mice did not differ in traveling latency and distance to reach the escape platform during the training phase (C,D; two-way ANOVA, *p* > 0.05, *n* = 17, 5 and 14 for tau(wt)/Nlrp3(wt), tau(tg)/Nlrp3(wt) and tau(tg)/Nlrp3(ko) mice, respectively); In the probe trial, tau(tg)/Nlrp3(wt) mice visited the region where the platform was previously located significantly less frequently than the tau(wt)/Nlrp3(wt) mice, and deficiency of NLRP3 in tau(tg)/Nlrp3(ko) mice significantly improved the memory of tau mice, as shown by the increased frequency of visiting the platform region compared to tau(tg)/Nlrp3(wt) mice (E; one-way ANOVA followed by Bonferroni *post-hoc* test).

Anyway, deficiency of NLRP3 reduced the number of AT8-positive neurons in the hippocampus, indicating the attenuation of tauopathy, in tau-transgenic mice (Figures 2A,B; one-way ANOVA, *p* < 0.001). In Morris water maze test, the traveling latency and distance before reaching the platform in the training phase did not differ between tau-transgenic and wild-type mice, and between tau-transgenic mice with and without knockout of *Nlrp3* gene, indicating that all our mice had comparable learning ability in spatial exploration (Figures 2C,D; two-way ANOVA, *p* > 0.05). Notably, the probe trial testing the memory of mice showed that tau-transgenic mice crossed the region where the original platform was located markedly less frequently than tau wild-type mice (Figure 2E; one-way ANOVA, *p* < 0.05), as we observed in a previous study (Qin et al., 2016). Notably, knockout of *Nlrp3* gene significantly reversed this memory impairment (Figure 2E; one-way ANOVA, *p* < 0.05).

### NLRP3 deficiency prevents the decline of cerebral vasculature and pericytes in tau-transgenic mice

**Figure 3:**
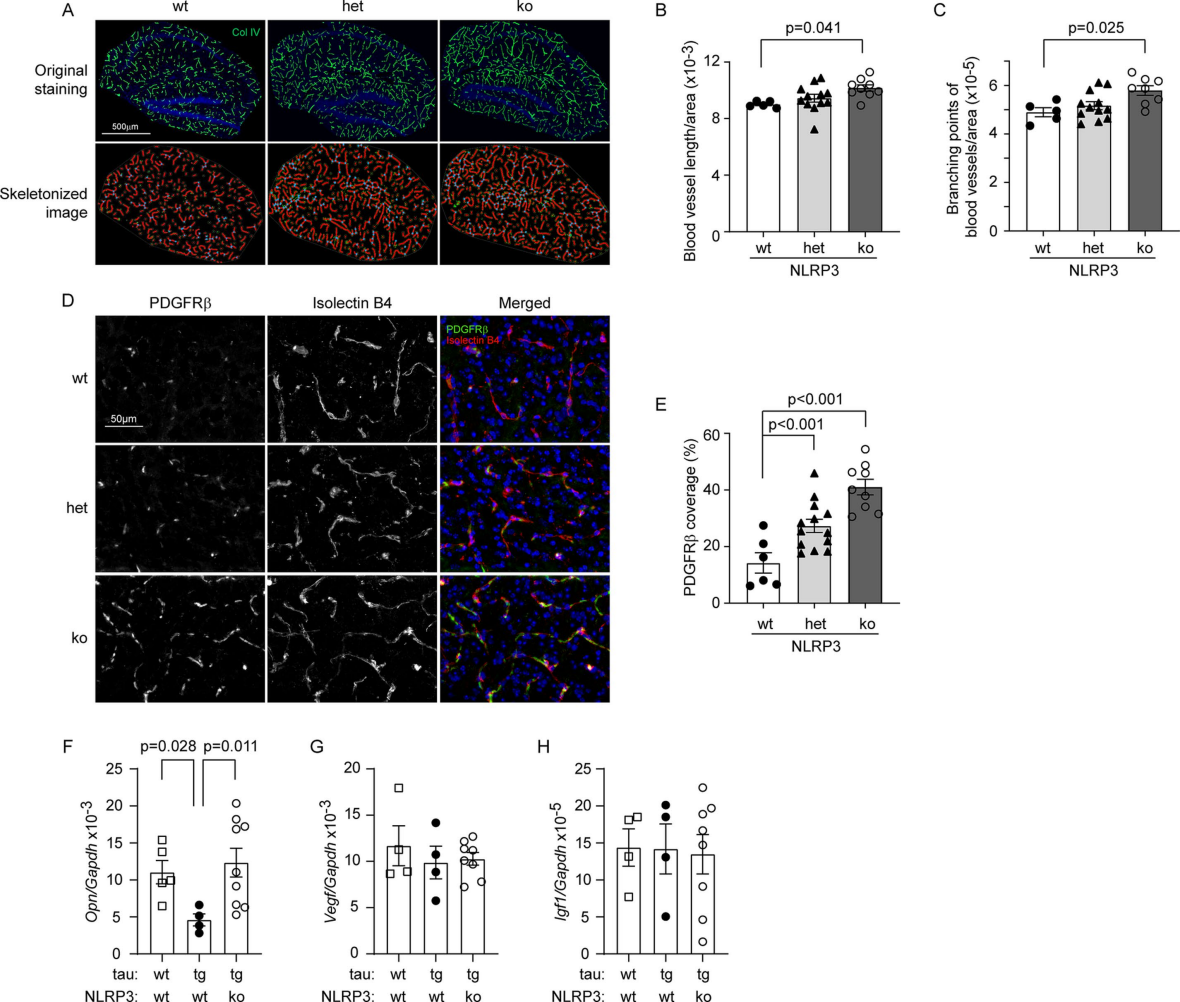
Deficiency of NLRP3 increases vasculature and pericyte coverage, and up-regulates *Osteopontin* gene transcription in the brain of tau-transgenic mice. The brains of 9-month-old tau-transgenic mice with homozygous (ko) and heterozygous (het) knockout, and wild-type (wt) of *Nlrp3* gene were stained for collagen type IV (Col IV) (A). The blood vessels in the hippocampus were thresholded and skeletonized. The skeleton representation of vasculature is shown in red and branching points of blood vessels are in blue (A). Deficiency of NLRP3 significantly increased the total length and branching points of blood vessels after adjusted by area of analysis in a gene dose-dependent manner (B,C; One-way ANOVA followed by Bonferroni *post hoc* test, *n* = 5, 12, and 9 for NLRP3 [wt, het and ko] mice, respectively). Brain tissues were further stained for pericytes with PDGFRβ antibody and vessels with isolectin B4 (D). Deficiency of NLRP3 significantly increased the ratio of PDGFRβ-immunoreactive and isolectin B4-stained area with a gene dose-dependent pattern (E; One-way ANOVA followed by Bonferroni *post hoc* test, *n* = 5, 13, and 9 for NLRP3 [wt, het and ko] mice, respectively). The transcription of angiogenesis-related genes *Osteopontin (Opn)*, *Vegf* and *Igf1*, was measured in the brains of tau mice using real-time PCR (F–H). Overexpression of tau protein significantly reduced the transcription of *Opn* and knockout of *Nlrp3* gene reversed it (F; one-way ANOVA followed by Bonferroni *post-hoc* test, *n* = 4–9 per group). Transcription of *Vegf* and *Igf1* was not altered by tau expression and knockout of *Nlrp3* gene (G,H; one-way ANOVA, *n* = 4–8 per group).

In our previous study, we had observed that the vasculature was reduced in 8-month-old tau-transgenic mice compared to wild-type littermates (Decker et al., 2018). After successfully establishing NLRP3-deficient and wild-type tau-transgenic mice, we stained brain tissues with antibodies against collagen type IV and compared the vasculature between different groups of tau mice with wild-type, heterozygous and homozygous knockout of *Nlrp3* gene. As shown in Figures 3A–C, knockout of *Nlrp3* gene increased both the length and branches of microvessels in the hippocampus of tau mice in a gene dose-dependent manner (one-way ANOVA, *p* < 0.05). Similarly, we co-stained pericytes with PDGFRβ antibody and blood vessels with isolectin B4, and clearly found that knockout of *Nlrp3* gene increased the coverage of PDGFRβ-positive pericytes on microvessels of the hippocampus of tau-transgenic mice also with a gene dose-dependent pattern (Figures 3D,E; one-way ANOVA, *p* < 0.05).

Recently, we observed that haploinsufficiency of microglial MyD88 reserves vasculature in APP/PS1-transgenic mice in association with upregulated expression of *Opn* and *Igf1* genes in microglia (Quan et al., 2021). We measured transcripts of *Opn*, *Igf1* and *Vegf* genes in brains of NLRP3-deficient and wild-type tau-transgenic mice, as well as tau wild-type mice. Transgenic expression of tau protein significantly reduced the transcription of *Opn* gene compared to wild-type mice (Figure 3F; one-way ANOVA, *p* < 0.05). Interestingly, knockout of *Nlrp3* gene reversed the reduction of *Opn* transcription in tau-transgenic mice (Figure 3F; one-way ANOVA, *p* < 0.05). Neither knockout of *Nlrp3* gene nor overexpression of tau altered the transcription of *Igf1* and *Vegf* genes compared to the corresponding controls (Figures 3G,H; one-way ANOVA, *p* > 0.05).

### NLRP3 deficiency protects pericytes from IL-1β challenge

**Figure 4:**
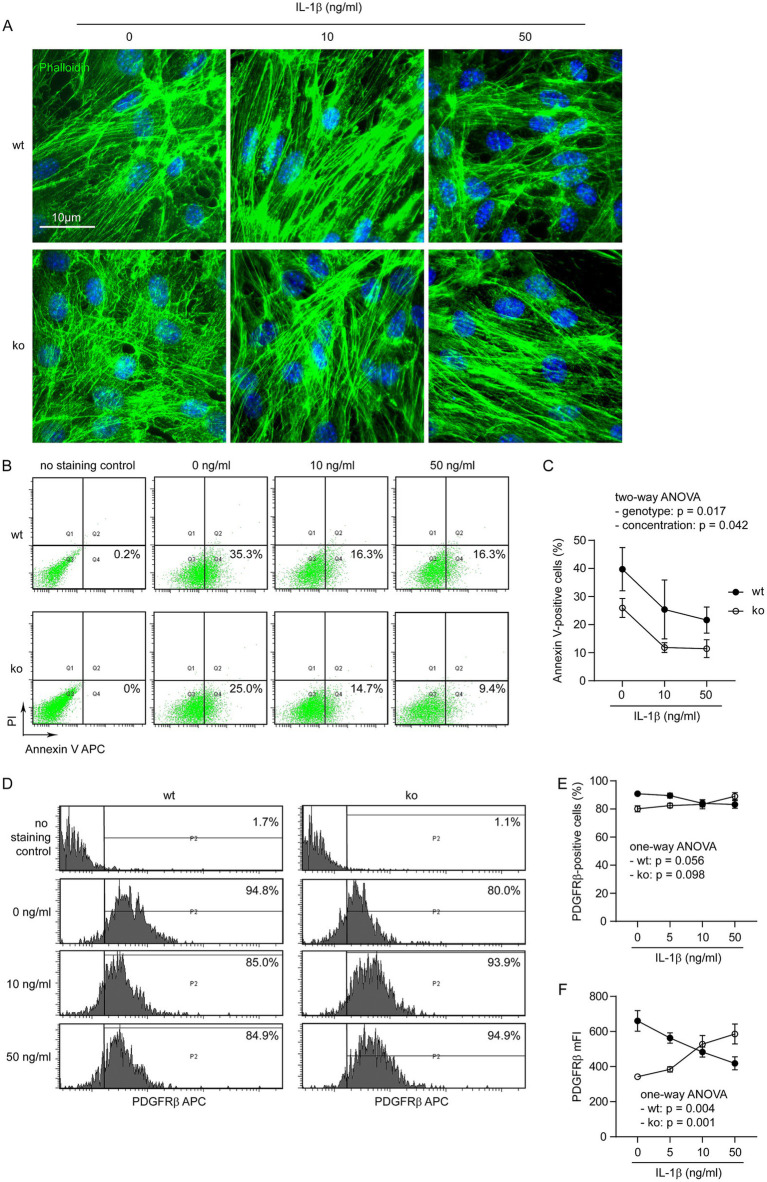
IL-1β increases PDGFRβ expression in NLRP3-deficient pericytes but decreases it in NLRP3 wild-type cells. Pericytes were cultured from brains of *Nlrp3*-knockout (ko) and wild-type (wt) mice and treated with IL-1β at 0, 5, 10 and 50 ng/mL for 24 h. Pericytes were then imaged after staining with fluorescent phalloidin (A), or detached for staining with APC-conjugated annexin V and propidium iodide (PI) (B), or APC-conjugated PDGFRβ antibody (D) and then analyzed by flow cytometry. NLRP3 deficiency and treatment of IL-1β synergistically reduced the percentages of annexin V-positive pericytes (C; two-way ANOVA; *n* = 3–4 independent experiments). NLRP3 deficiency and IL-1β treatment did not alter the percentage of PDGFRβ-expressing cells (E; two-way ANOVA; *p* > 0.05, *n* = 5 independent experiments). Of note, IL-1β treatment significantly reduced mFI of PDGFRβ in NLRP3 wt pericytes, while it increased the mFI of PDGFRβ in NLRP3 ko cells, both in a dose-dependent manner (F; one-way ANOVA for NLRP3 wt and ko cells, separately, *n* = 5 independent experiments).

Pericytes are essential for the development of vasculature (Montagne et al., 2018). We wanted to know whether NLRP3-deficient and wild-type pericytes respond differently to inflammatory challenges in the AD brain. Due to technical difficulties in our laboratory, we were unable to specifically knock out *Nlrp3* gene in pericytes in the tau-transgenic mouse brain. Since IL-1β is an important inflammatory cytokine that is overexpressed in AD brain (Mrak and Griffin, 2000), we treated cultured *Nlrp3*-knockout and wild-type primary pericytes with recombinant IL-1β at 0, 5, 10 and 50 ng/mL as we did in the human pericyte line (Quan et al., 2020). Under microscope, we could see a well-organized phalloidin-stained actin cytoskeleton with clear and distinct filamentous structures, except very few 50 ng/mL IL-1β-treated NLRP3 wild-type cells showing irregular and dispersed phalloidin staining and cell shrinkage (Figure 4A). We did not observe typical morphology of pyroptosis, such as chromatin condensation, intact nuclei, cellular swelling, and plasma-membrane rupture (Bergsbaken et al., 2009). In order to examine cell death in the brain, we also performed immunohistochemistry with antibodies against cleaved caspase-3, we could not observe any immunoreactive cells in the brains of tau mice with and without knockout of *Nlrp3* gene (data not shown).

In following experiments, we stained cells with fluorescence-conjugated annexin V and observed that treatments of IL-1β decreases apoptotic cells in both NLRP3-deficient and wild-type pericytes (Figures 4B,C; two-way ANOVA, *p* < 0.05), which was consistent with our previous finding (Quan et al., 2020). Notably, deficiency of NLRP3 significantly reduced annexin V-stained pericytes (Figures 4B,C; two-way ANOVA, *p* < 0.05).

Thereafter, we measured PDGFRβ expression on pericytes with flow cytometry. The binding of endothelial cells-released PDGF-B to PDGFRβ on pericytes is essential for proliferation and integration of pericytes in the cerebral vasculature (Lindahl et al., 1997). Treatment with IL-1β significantly decreased PDGFRβ expression in *Nlrp3* wild-type cells but increased it in *Nlrp3*-knocked out pericytes as shown by changes in mean fluorescence intensity (mFI; Figures 4D,F; one-way ANOVA analysis separately for *Nlrp3*-wildtype and knockout cells, *p* < 0.05). Perhaps due to a high expression level of PDGFRβ in our cultured pericytes, we only observed a tendency of IL-1β-induced changes in the percentages of PDGFRβ-positive NLRP3-deficient and wild-type cells (Figures 4D,E; separate one-way ANOVA analysis for *Nlrp3*-wildtype and knockout cells, 0.05 < *p* < 0.01).

### NLRP3 deficiency protects pericytes from hydrogen peroxide challenge

**Figure 5:**
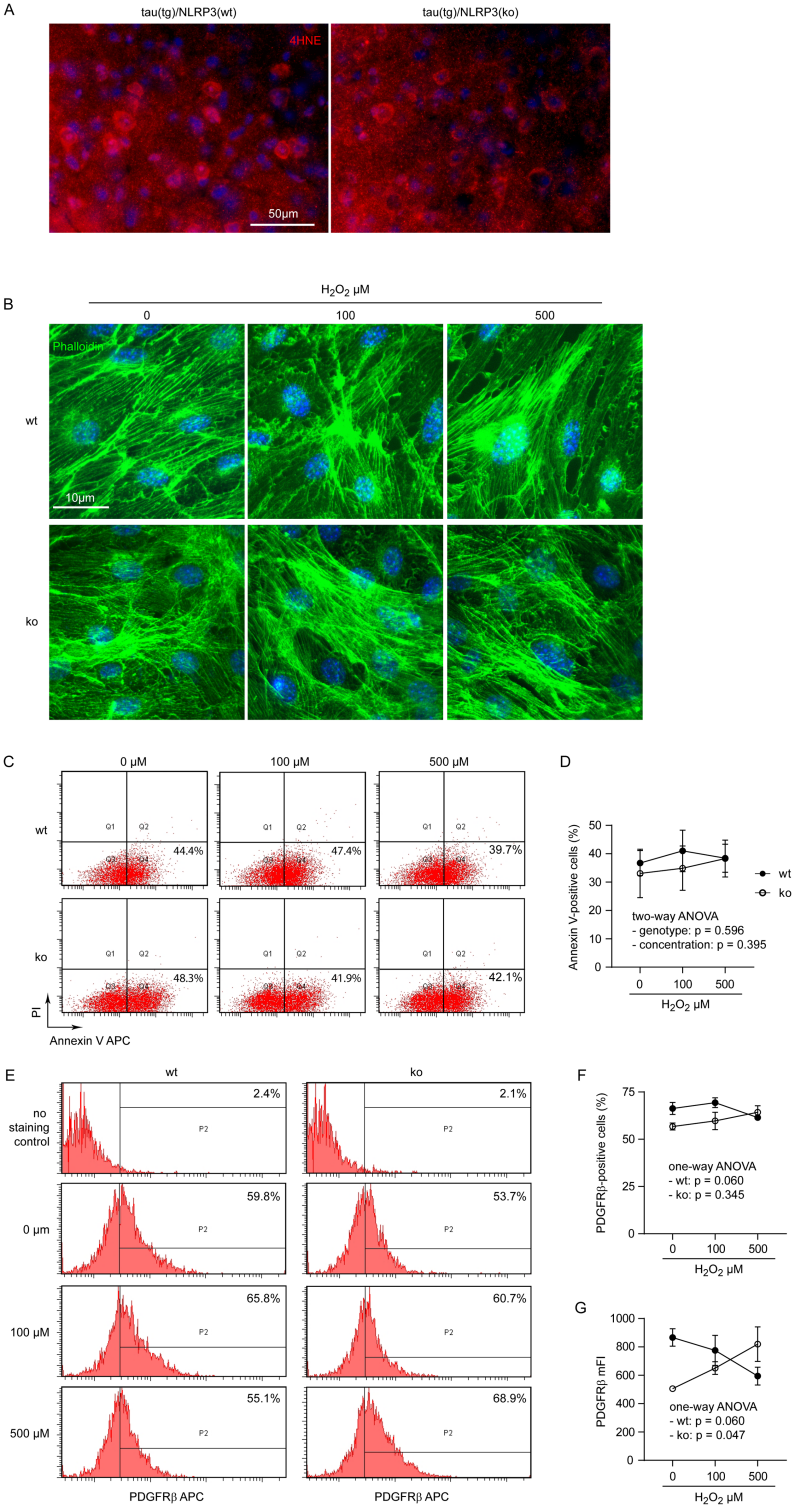
H_2_O_2_ increases PDGFRβ expression in NLRP3-deficient but not wild-type pericytes. Brain sections from 9-month-old tau^tg^Nlrp3^+/+^ (NLRP3 wt) and tau^tg^Nlrp3^−/−^ (NLRP3 ko) mice were stained with 4HNE antibody for evidence of oxidative modification of brain tissue in tau mice (A). Pericytes were then cultured from brains of *Nlrp3*-knockout (ko) and wild-type (wt) mice and treated with H_2_O_2_ at 0, 100 and 500 μM for 24 h. Pericytes were imaged after staining with Alexa488-conjugated phalloidin (B), or detached for staining with APC-conjugated annexin V and propidium iodide (PI) (C), or APC-conjugated PDGFRβ antibody (E) and then analyzed by flow cytometry. NLRP3 deficiency and treatment of H_2_O_2_ did not change the percentages of annexin V-positive pericytes (D; two-way ANOVA; *n* = 3–6 independent experiments). NLRP3 deficiency and H_2_O_2_ treatment did not alter the percentage of PDGFRβ-expressing cells (F; two-way ANOVA; *p* > 0.05, *n* = 4–7 independent experiments). Interestingly, H_2_O_2_ treatment significantly increased mFI of PDGFRβ in NLRP3 ko cells, but tended to reduce it in NLRP3 wt pericytes, both in a dose-dependent manner (G; one-way ANOVA for NLRP3 wt and ko cells, separately, *n* = 4–7 independent experiments).

We detected brain cells with distinct staining of the 4HNE antibody, indicating lipid peroxidation of the cells, although it was difficult to quantitate the fluorescence intensity of the stained cells in NLRP3-deficient and wild-type tau transgenic mice (Figure 5A). Hydrogen peroxide (H_2_O_2_) is an important inflammatory mediator in AD brain (Chun et al., 2020). We additionally treated *Nlrp3*-knockout and wild-type primary cultured pericytes with H_2_O_2_ at 0, 100 and 500 μM. Challenges of H_2_O_2_ changed neither the cell skeletons (Figure 5B), nor percentages of annexin V-stained pericytes in both NLRP3-deficient and wild-type pericytes (Figures 5C,D; two-way ANOVA, *p* > 0.05). Similarly, treatments with H_2_O_2_ significantly increased expression of PDGFRβ in NLRP3-knockout pericytes in a dose-dependent manner, but tended to decrease PDGFRβ expression in NLRP3 wild-type cells (Figures 5E,G; separate one-way ANOVA analysis for NLRP3-wildtype and knockout cells). It was the same as IL-1β treatment that H_2_O_2_ did not significantly change the percentages of PDGFRβ-positive cells among NLRP3-deficient and wild-type pericytes (Figures 5E,F; one-way ANOVA analysis for NLRP3-wildtype and knockout cells, *p* > 0.05).

## Discussion

Growing evidence implicates vascular dysfunction in AD pathogenesis (Strickland, 2018). By cross-breeding tau-transgenic mice and *Nlrp3*-knockout mice, we demonstrated that NLRP3 deficiency reserves cerebral pericytes and vasculature in tau-transgenic mice, which may in turn attenuate tauopathy and improve cognitive function. As a possible mechanism, NLRP3 deficiency reduces pericyte apoptosis and maintains pericyte expression of PDGFRβ against the inflammatory challenge.

Our previous study has shown that the vasculature is reduced in the brain of tau-transgenic mice (Decker et al., 2018). In another study, the vascular volume detected by perfusion of cerebral arteries with [14C]-sucrose is reduced in 3 × Tg-AD, which overexpress human APPSwe, presenilin-1 (PS1M146V), and tau (tauP301L), but not in APP/PS1-transgenic mice, indicating the role of p-tau in vascular injury (Do et al., 2014). Since we also observed that cerebral vasculature is reduced in APP/PS1-transgenic mice (Decker et al., 2018), which is reversed by deletion of MyD88 in microglia (Quan et al., 2021). We hypothesized that the NLRP3-mediated modification of inflammatory activation may affect the vasculature in tau mice. Indeed, in the mouse model of oxygen-induced ischemic retinopathy, treatment with an NLRP3 inhibitor decreases IL-1β expression and increases pericyte density, which reduces acellular capillaries and retinal vessel leakage (Sui et al., 2020). Although we did not detect IL-1β protein levels in our NLRP3-deficient tau mice, there should be no doubt that IL-1β secretion was reduced because NLRP3-containing inflammasome is essential for the production of active IL-1β from its precursor protein (Jiang et al., 2021; Luciunaite et al., 2020). In both our microglial MyD88-deficient APP/PS1 transgenic mice (Quan et al., 2021) and the current NLRP3-deficient tau-transgenic mice, cerebral vasculature reservation correlates with upregulation of *Opn* gene transcription. Osteopontin, a secreted inflammatory cytokine, is strongly upregulated during ischemia. It promotes the recruitment of inflammatory cells, e.g., macrophages, and is required for post-ischemic neovascularization, including cell survival, adhesion, migration and proliferation. However, a chronic increase in osteopontin promotes atherosclerosis and decreases anti-inflammatory atheroprotective cytokines, e.g., IL-10 (Lok and Lyle, 2019). In AD, the pathophysiological function of osteopontin for cerebrovascular homeostasis needs to be investigated.

However, the inflammatory modification in NLRP3-deficient tau-transgenic mice is complicated, as the transcription of inflammatory mediators, including TNF-*α* and IL-1β, was not altered by deficiency of NLRP3 in brain tissue. Recently, there were two studies showing that knockout of *Nlrp3* gene did not alter inflammatory activation of microglia in APP/PS1-transgenic mice (Srinivasan et al., 2024), nor did it reduce the IL-18 cytokine in plasma in P301S-tau transgenic mice (Paesmans et al., 2024). Similar to IL-1β, biologically active IL-18 is generated intracellularly from its precursor protein by caspase 1 in NLRP3 inflammasome (Gu et al., 1997). Since we observed that NLRP3 deficiency decreases cerebral vasculature under physiological conditions, where inflammatory activation is at a low level (Quan et al., 2020), we proposed that there are mechanisms other than inflammation that mediate the effects of NLRP3 in maintaining vasculature and pericytes in tau mice, e.g., the protective effect of NLRP3 deficiency on blood vessels may also be due to the increased resistance of pericytes to inflammatory damage.

NLRP3-contained inflammasome cleaves pro-caspase-1 into its active form, caspase-1, which may cause pyroptosis (Tsuchiya, 2020). However, we did not observe any morphology characteristic of pyroptosis in our cultured pericytes after treatment with Il-1β and H_2_O_2_. There was an apoptotic cell population (30%) stained by annexin-V in our cultured pericytes, which could be drastically reduced by IL-1β treatment. Although IL-1β has been shown to induce apoptosis of pericytes under high glucose conditions (Yun, 2021), IL-1β also prevents apoptosis of mature neutrophils, in which apoptosis is constitutively active (William et al., 1998). Remarkably, deficiency of NLRP3 prevented apoptosis of our cultured pericytes independent of IL-1β treatment. The anti-apoptotic mechanism of NLRP3 inhibition is unclear. There is evidence that suppression of NLRP3-inflammasome activity reduces both apoptosis and pyroptosis of mesenchymal stromal cells (MSC) in three-dimensional culture and improves the therapeutic efficacy of transplanted MSC spheroids in a mouse model of colitis (Pham et al., 2023).

PDGF-B, which is released by endothelial cells, binds to PDGFRβ on pericytes and promotes proliferation and integration of pericytes into cerebral vasculature (Lindahl et al., 1997). In a tumor study, elimination of pericyte induces apoptosis of endothelial cells (Song et al., 2005). These studies suggest an essential role of PDGF-B and PDGFRβ in the maintenance of pericyte and vascular homeostasis in the brain. Indeed, impairment of PDGF-B and PDGFRβ signaling leads to structural and functional defects of capillaries in the adult mouse brain (Bell et al., 2010). Our previous experiments in immortalized human pericytes have shown that: (1) transient treatment with IL-1β increases the expression of PDGFRβ, which is reversed by AKT inhibition (Quan et al., 2020); and (2) long-term treatment with IL-1β at a high concentration (50 ng/mL) tends to decrease PDGFRβ expression (Quan et al., 2021). In the present study, we observed that transient treatment with IL-1β or H_2_O_2_ decreased the expression of PDGFRβ in NLRP3 wild-type pericytes, but increases it in NLRP3-deficient cells. Therefore, the regulation of NLRP3 in PDGFRβ expression may depend on the state of the cell. Deficiency of NLRP3 confers better survival to pericytes, even similar to immortalized pericytes, although the underlying mechanism is unknown. Obviously, it is difficult to compare the conditions in cell culture with those in the brain; our *in vitro* results are consistent with the results in the tau-transgenic mouse brain.

Our work has several limitations due to the technical difficulties: (1) we were unable to specifically knock out the *Nlrp3* gene in pericytes and analyze the distinct pathogenic effect of NLRP3 in pericytes in tau-transgenic mice. In our current NLRP3-deficient tau mice, the potential reduction of IL-1β secretion and the improvement of pericyte resistance to inflammatory challenges cannot be distinguished in case NLRP3 inhibition leads to AD prevention; (2) we missed the functional detection of blood flow in the brain of tau-transgenic mice. Blood flow is important for the regulation of neuronal activity; (3) we quantified the length and branching of microvessels but did not analyze the morphology of vessels. Tg4510 tau-transgenic mice have been reported to have more capillaries, but with atypical and spiral morphology and reduced luminal diameter of blood vessels (Bennett et al., 2018); and (4) we failed to analyze pericyte death and proliferation in the brain of tau-transgenic mice. This could be due to the very slow process of these two events.

In summary, deficiency of NLRP3 not only reduces neuroinflammation, but also increases PDGFRβ expression and prevents apoptosis in pericytes. Deficiency of NLRP3 promotes vascular homeostasis, attenuates tauopathy and improves cognitive function in tau-transgenic mice. Our study supports clinical trials with NLRP3 inhibitors for the prevention/treatment of AD patients. However, the potential loss of physiological effect of NLRP3 on vascular maintenance in the relatively healthy brain region should be considered as a side-effect in future translational studies of NLRP3 inhibitors in AD patients (Quan et al., 2020).

## Data Availability

The original contributions presented in the study are included in the article, further inquiries can be directed to the corresponding author.

## Footnotes

^1^http://angiotool.nci.nih.gov

^2^https://imagej.nih.gov/ij/
